# For a New University

**Published:** 1907-05

**Authors:** 


### For a New University.

Vice-Chancellor Charles P. Norton, of the University of Buffalo, and a member of the committee in charge of the university extension movement, appeared before the almshouse committee of the board of supervisors on April 12, and made a formal offer to exchange a farm of between 500 and 700 acres for the county property on Main street at the city line on which stand the almshouse and the county hospital.

The committee listened to Mr. Norton's address with considerable interest as it dawned on them that this was the first definite public step toward the establishment of a university liberal arts department. While there has not been any reply made with regard to the exchange so far as relates to location of the farm offered, the supervisor's committee has been given the choice of several farms of the required size on which the university authorities have options.

The plan is that as soon as the county property at the city line is secured as a site for the new university, application will be made to the Rockefeller fund trustees, with every prospect that such an application for endowment will be successful.

